# Micro-Aqueous Organic System: A Neglected Model in Computational Lipase Design?

**DOI:** 10.3390/biom11060848

**Published:** 2021-06-07

**Authors:** Shang Wang, Yan Xu, Xiao-Wei Yu

**Affiliations:** Key Laboratory of Industrial Biotechnology, Ministry of Education, School of Biotechnology, Jiangnan University, Wuxi 214122, China; wangshangedu@foxmail.com (S.W.); yxu@jiangnan.edu.cn (Y.X.)

**Keywords:** lipase, molecular dynamic simulation, non-aqueous phase catalysis

## Abstract

Water content is an important factor in lipase-catalyzed reactions in organic media but is frequently ignored in the study of lipases by molecular dynamics (MD) simulation. In this study, *Candida antarctica* lipase B, *Candida rugosa* lipase and *Rhizopus chinensis* lipase were used as research models to explore the mechanisms of lipase in micro-aqueous organic solvent (MAOS) media. MD simulations indicated that lipases in MAOS systems showed unique conformations distinguished from those seen in non-aqueous organic solvent systems. The position of water molecules aggregated on the protein surface in MAOS media is the major determinant of the unique conformations of lipases and particularly impacts the distribution of hydrophilic and hydrophobic amino acids on the lipase surface. Additionally, two maxima were observed in the water-lipase radial distribution function in MAOS systems, implying the formation of two water shells around lipase in these systems. The energy landscapes of lipases along solvent accessible areas of catalytic residues and the minimum energy path indicated the dynamic open states of lipases in MAOS systems differ from those in other solvent environments. This study confirmed the necessity of considering the influence of the microenvironment on MD simulations of lipase-catalyzed reactions in organic media.

## 1. Introduction

Lipases (triacylglycerol acyl hydrolases, EC 3.1.1.3) catalyze the synthesis and hydrolysis of ester bonds of lipids [1,2]. Lipases are widely applied in the bioenergy, pharmaceutical, detergent and food industries [3,4]. Lipases have received attention from researchers due to their wide substrate scope, ease of handling and high stability to various temperatures and solvents.

Enzymatic transformations in organic solvents are of interest in lipase studies. Unwanted side reactions such as polymerization, hydrolysis, racemization and decomposition often occur in water, which limits many of the reactions of interest in enzymatic synthesis. Moreover, nonpolar compounds exhibit good solubility in some organic solvents. Most of the synthetic reactions run on an industrial scale can be carried out in organic solvents. Enzymatic studies of lipases now intersect a variety of disciplines, including structural biology, molecular biology, computational biology, etc. Of these, computational simulations, especially molecular dynamics (MD) simulations, are becoming widely used tools in the study of enzymatic transformations in organic solvents. Researchers are contributing greatly to insights into the effects of organic solvents on lipases. Sanker et al. [5] performed MD simulation with plant lipase to study the lid changes of lipase in toluene solvent. Zhu et al. [6] investigated the conformational stability of porcine pancreatic lipase in three non-aqueous organic solvents, including dimethyl sulfoxide, propylene glycol and ethanol, through MD simulation. Mohtashami et al. [7] investigated the structural behaviors of enzymes with completely different structural architectures, including lipase, laccase and lysozyme, in the presence of methanol and hexane. The conformational changes of Yarrowia lipolytica lipase in organic solvents were also investigated by MD [8]. However, enzymatic transformations in organic solvents are often affected by small amounts of water contained in the solvent [9,10] and this system is a so-called micro-aqueous organic solvent (MAOS). Related MD studies have frequently ignored the possible effects of a small amount of water on the lipase in organic solvents. Although this might seem trivial, it is easy to underestimate its importance.

Water content (even a small amount of water content) is an important factor in lipase-catalyzed reactions. The effect of the organic solvent on the enzyme is primarily due to interactions with the enzyme-bound, essential layer of water rather than with the enzyme itself [11]. The water required by enzymes in non-aqueous solvents provides them with sufficient conformational flexibility for catalysis [11]. In a previous study, MD simulations of MAOS-like systems (224 water:2289 chloroform, mol/mol) were performed by Pleiss et al. [12]. However, the previous study was limited by the computational resources available at the time and only short time scale MD simulations were performed. The MAOS system was not the focus of the previous study [12] and was not discussed further. We hope to construct a model in a MD simulation to demonstrate the conformational differences of lipases existing between the MAOS system and non-aqueous organic solvent systems; this, the authors believe, will provide additional insights into our understanding of lipase catalysis in organic systems.

In this study, several commonly used models in the lipase catalysis industry were selected, including *Candida antarctica* lipase B (CALB), *Candida rugosa* lipase (CRL) and *Rhizopus chinensis* lipase (RCL), as models for studies of MD simulations run under water, non-aqueous organic solvent (n-heptane or toluene) and MAOS solvent conditions. The uniqueness of lipases in MAOS media is mainly elucidated from the following three factors: (i) The conformational differences of lipases in various systems. (ii) The dynamic behavior of water in the MAOS systems and ramifications for the protein surface. (iii) The different dynamic open states of lipases in MAOS systems as distinguished from those in other solvent environments. Our study differs from previous MD studies in that our focus is on the effects of MAOS systems on lipases. This study confirms the necessity of considering the influence of a small amount of water in MD simulations of lipase-catalyzed reactions in organic media.

## 2. Methods

### 2.1. System Setup and MD Simulations

The models of CALB, CRL and RCL were based on data from the Protein Data Bank (https://www.rcsb.org, accessed on 10 March 2021) entries 1TCA, 1TRH and 6A0W, respectively. All three of these enzymes exhibited ester synthesis activity. In these X-ray crystal structures, the active pockets are covered by different types of lids. (CALB is excluded because it is classified as a lidless lipase [13].) The tautomeric and protonation states of residues at pH 7.0 were assigned with PROPKA [14]. AmberTools18 [15] was used to generate n-heptane and toluene parameter files and topological files for the simulations. The coordinates of n-heptane molecule and toluene molecule were obtained from PubChem (http://pubchem.ncbi.nlm.nih.gov, accessed on 10 March 2021, CID 8900 and CID 1140). The molecular structures of n-heptane and toluene were optimized with Gaussian 16 [16]. The calculation level for structural optimization was B3LYP (D3)/6-31G** [17,18,19]. The single-point energy was calculated at the M06-2X (D3)/def2-TZVP [20,21] calculation level. The polarizable continuum model (PCM) [22] was used to show the water environment in the calculation of the n-heptane and toluene optimization and the Solvation Model Based on Density (SMD) [23] implicit solvent model was used to show the water environment in the substrate molecule single point energy calculation. The restrained electrostatic potential (RESP) [24] charge was generated using Multiwfn 3.7 [25].

GROMACS Package [26] (version 2019.6) was used to perform all MD simulations. The generalized AMBER force field [27] (GAFF) and AMBER14SB+parmbsc1 [28] were used to simulate all systems. In addition, disulfide bond restraints were generated for the CALB disulfide bonds (Cys22- Cys64, Cys216- Cys258, Cys293- Cys311), CRL disulfide bonds (Cys60- Cys97, Cys268- Cys277) and RCL disulfide bonds (Cys56-Cys295, Cys67-Cys70, Cys262-Cys271), respectively. MD simulations were performed under five conditions. One simulation condition was referred to as the aqueous system, in which a cubic simulation box filled with water was placed around the lipase within a distance of 13 Å. The second simulation condition was referred to as the non-aqueous organic solvent system, in which a cubic simulation box filled with n-heptane or toluene molecules was placed around the lipase within a distance of 13 Å. The densities of the non-aqueous organic solvent system were calculated after energy minimization, heating and equilibration. The results were in good agreement with the experimental densities, as shown in Table S1. The last simulation condition was referred to as the MAOS system, in which lipase was placed in a cubic simulation box filled with 600 water molecules and 2039 n-heptane molecules (MAOS_hep_) or 413 water molecules and 2000 toluene molecules (MAOS_tol_) used to represent the MAOS media (water content 3.5%, *v*/*v*). Periodic boundary conditions were set in the x, y and z directions. Neutralization of the system was carried out by replacing water molecules with Na+ and Cl− ions. The steepest descent energy minimization was used in the first 500 steps and the conjugate gradient method was used in the last 2000 steps. After energy minimization, 2 ns of NVT equilibration at 313 K was performed. Bond length constraints were applied to H-bonds based on LINCS [29]. Long-range electrostatic effects were handled using PME [30] and temperature coupling was performed using velocity rescaling with a stochastic term. Position restraints with a force constant of 10.0 kcal/mol/Å^2^ were imposed on lipase during the heating steps. After NVT equilibration, 5 ns of NPT equilibration at 313 K was performed. Berendsen’s method was used for pressure coupling. The cutoff distance for van der Waals interactions was 10.0 Å. After NPT equilibration, 200 ns production simulations were performed on all systems. The Parrinello–Rahman method was used for pressure coupling.

### 2.2. Analytical Methods

Snapshots of the backbones of the lipase were obtained by VMD [31]. During the 200 ns simulation, 20 frames of the lipase conformations were extracted evenly and the translations and rotations of the trajectory were removed. The blue-green-red color bar represents 0 ns to 200 ns. The protein is displayed in NewRibbons mode and the material is set to transparent.

The structure root-mean-square deviation (RMSD) of backbone atoms (C, CA and N atoms of each residue) was calculated with the program gmx_rms. The root-mean-square fluctuation (RMSF) of backbone atoms was calculated with the program gmx_rmsf.

Solvent accessible surface areas (SASA), hydrophilic surface area (SAphilic) and hydrophobic surface area (SAphobic) were calculated using the DSSP algorithm [32,33].

Snapshots of the waters in the three lipase MAOS systems at different instants were obtained by VMD. During the 200 ns simulation, 10 frames of the water in various conformations were extracted evenly and the translations and rotations of the trajectory were removed. The blue-green-red color bar represents 0 ns to 200 ns. The water is displayed in line mode and the material is set to transparent. Cartoon models of lipase with different colors represent different secondary structures. Purple, blue, red, yellow, cyan and white parts represent alpha-helix, 3/10 helix, pi_helix, beta-sheet, turn and coil, respectively.

Energy E(x) was calculated through the Boltzmann distribution. Energies were expressed in kT units, where k is the Boltzmann constant and T is the absolute temperature, which was taken as 313 K. Energy landscape (EL) analysis was conducted according to the probability density P(x). Each trajectory was sampled every 100 ps in the EL analysis.

The point with the highest energy along the minimum energy path (MEP) is the transition state (TS). It is a first-order saddle point on the energy surface. MEP was plotting using MEPplot based on string method [34].

VMD 1.9.3 [31] software was used for visual inspection of trajectories. The figures and videos of enzymes were rendered by the Tachyon ray tracer (VMD software plugin).

### 2.3. Protein, Expression and Assay of Lipase Synthetic Activity

The expression and production of RCL followed previously described procedures [35]. Briefly, polymerase chain reaction-amplified gene products were inserted into the pPIC9K expression vector. Plasmids were electroporated into *Pichia pastoris* GS115 and expressed in BMGY and BMMY media at 30 °C. After induction at 28 °C for 86 h, the culture was centrifuged at 7000× *g* for 20 min. The protein concentration of the supernatant was diluted to 10 mg/mL. The supernatant was lyophilized for the assay of lipase synthetic activity.

Lipase synthetic activity was measured by ester-synthesis method in n-heptane according to the procedure described previously [18] with some modifications. Briefly, 3 mL substrate solution (3 M octanoic acid and ethanol in n-heptane, with acid/alcohol molar ratio of 1:1) was added in a 5 mL capped tube. All solvents used in this work were previously dried with 3 Å molecular sieves (20% *m*/*v*) for 120 h. According to the present study, this method could effectively remove water from organic solvents (reduce the water content to 8.2 ppm in ethanol [36]). The reaction was started by adding 20 mg lipase powder and was incubated for 30 min at 40 °C with a shaking speed of 220 rpm. The experiment was carried out by adding water to create a gradient at the water content of ~0%, 3.5%, 7%, 10%, 15%, 20% (*v*/*v*). Filtered using 0.22 μm membrane to remove the contaminants, samples (400 μL) were drawn and mixed with 100 μL of 2-hexanol, as internal standard, then analyzed by gas chromatography (GS). All the reactions were repeated for three times.

According to a previously described method [37], an Agilent 6890N gas chromatograph with a flame ionization detector (FID) was employed to quantitate the ester product catalyzed by lipase (ethyl octanoate). 1 μL of the sample was directly injected into the gas chromatograph in split mode (split ratio = 20:1). Nitrogen was used as the carrier gas at a constant flow rate of 1 mL/min and a CPWAX57CB column (30 m × 0.25 mm i.d., 0.25 μm film thickness) was used for separation. The column temperature was programmed as follows: initially at 35 °C for 1 min, increased by 3 °C/min to 70 °C, increased to 180 °C by 3.5 °C/min, increased by 15 °C/min to 210 °C and then held for 5 min. The injector and detector temperatures were set at 250 °C.

## 3. Results and Discussion

### 3.1. Overall Conformations of Lipases in Different Solvent Environments

**Figure 1:**
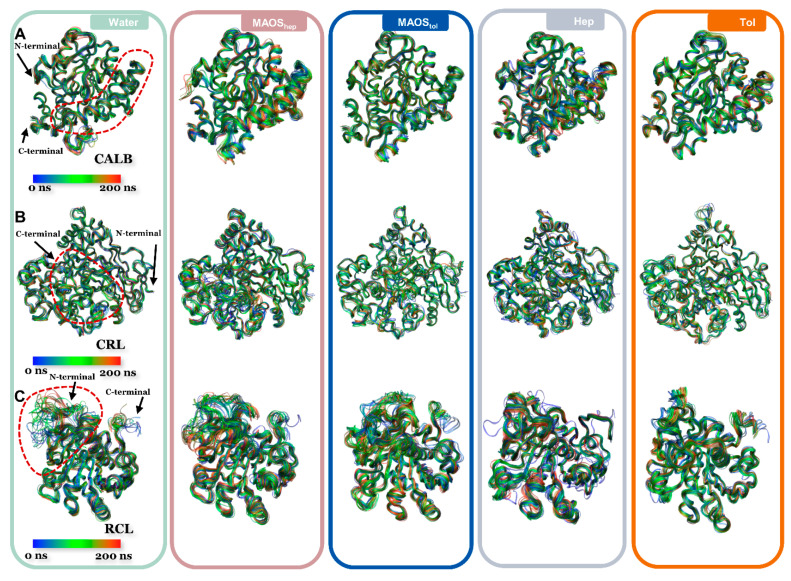
Snapshots of CALB backbones (**A**), CRL backbones (**B**) and RCL backbones (**C**) at different instants in water (green box), MAOS_hep_ (pinkbox), MAOS_tol _(blue box), n-heptane (ice-blue box), toluene (orange box). The time scale ranges from 0 ns to 200 ns and corresponds to blue-green-red colors. The regions denoted by red dashed lines represent the flexible regions observed during MD simulations in various solvent systems. Both terminus and the flexible regions of the lipases are only shown in the water system.

MD simulations were carried out on different systems, including the MAOS systems (600 water molecules, 2039 n-heptane molecules; 414 water molecules, 2000 toluene molecules; water content 3.5%, *v*/*v*), organic solvent (n-heptane or toluene) systems and aqueous systems. The water content of MAOS systems was determined by our previous experimental study [38]. It was speculated that the conformations of lipases in the MAOS systems were distinct from those of lipases in non-aqueous organic solvents and aqueous solution. To better understand the functional significance of the lipase structure in different solvent environments, it was necessary to map out the dynamics of the lipases in simulations. To achieve this, snapshots of the backbones of the three lipases at different instants are mapped in Figure 1. For the CALB systems, the lipase conformation in aqueous systems showed a relatively stable state and the lipase conformation in MAOS systems/non-aqueous organic systems exhibited a more flexible state. The flexible regions were concentrated in the dashed line sections in Figure 1A. For the CRL systems, the lipase conformations in aqueous systems showed a relatively stable state similar to that of CALB in aqueous systems. The CRL conformations of MAOS systems showed more flexible behavior than that of the aqueous system. For the RCL systems, the main differences in lipase conformations were concentrated at the N-terminal peptide region (red dashed line in Figure 1C). The direction of the N-terminal peptide perturbations produced in aqueous systems was from bottom to top. The N-terminal peptide perturbations in the MAOS_hep_ systems exhibited the opposite trajectory.

**Figure 2:**
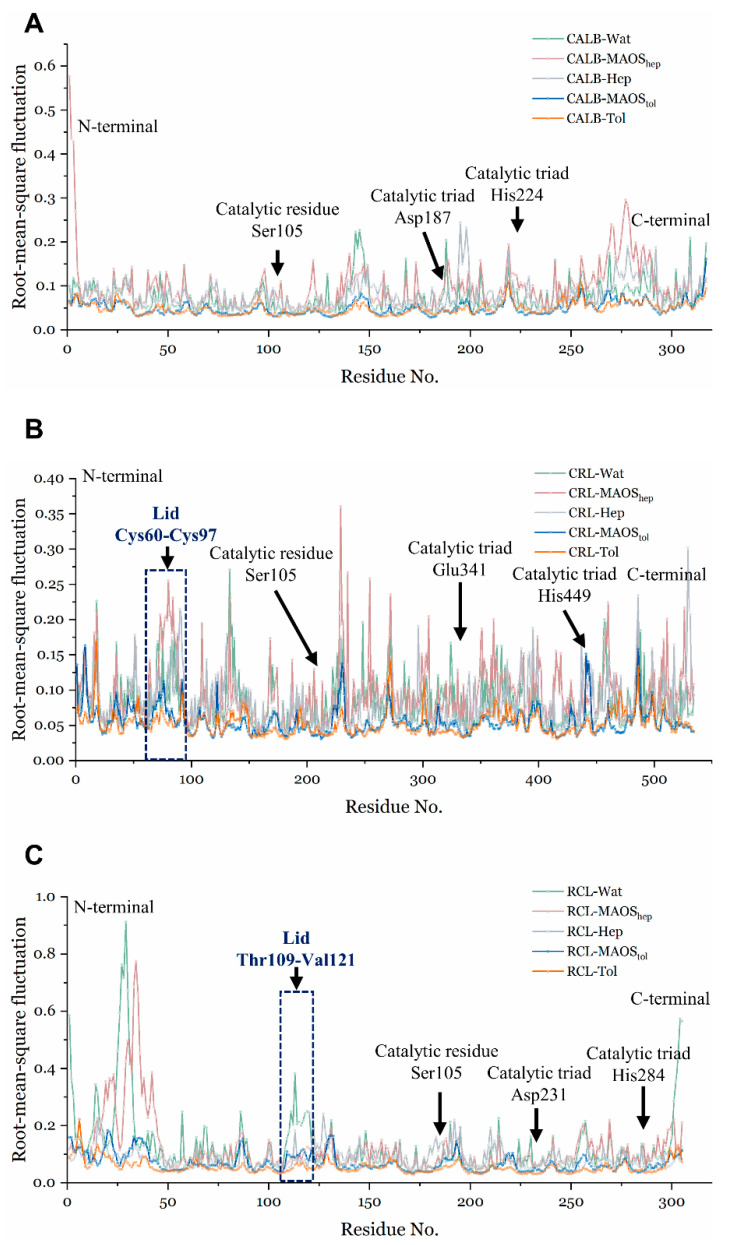
RMSF of CALB (**A**), CRL (**B**) and RCL (**C**) in the in water (green), MAOS_hep_ (pink), MAOS_tol_ (blue), n-heptane (ice-blue), toluene (orange). The lid region, catalytic residue and catalytic triad are presented in the corresponding regions.

The quantitative root-mean-square fluctuation (RMSF) was evaluated for the backbone atoms of lipases for all systems (Figure 2). For the CALB systems (Figure 2A), the region 185–193 (including catalytic triad Asp187) showed a flexible state in the water and MAOS_hep_ system. The region 220–228 showed a flexible state in MAOS_hep_. Meanwhile, region 270–285 of CALB close to the C-terminal showed a flexible state. The RMSF trends of the five different solvent environments were similar in the CRL system (Figure 2B). The CRL in MAOS_tol_ showed a more stable state than that in MAOS_hep_. Depending on the solvent polarity, the behavior of lipase in different MAOS systems might differ. Notably, the lid region of CRL showed differences from that in various solvent environments in their conformational flexibility. For the RCL systems, the major differences of RMSF existed in the N-terminal propeptide region. Following a previous study, the N-terminal propeptide of RCL plays an important role in its function [39]. Moreover, the lid region of RCL in the aqueous system exhibited a more flexible state. This difference in catalytic function of lipase under various solvent environments might arise from differences in the behavior of lid.

On the basis of these results from snapshots and RMSF, it was initially proposed that lipases in MAOS systems showed unique conformations distinct from those in other systems. However, only limited information could be obtained regarding this distinction. Some researchers contend that there are four factors determining how organic solvents might mediate an enzyme-catalyzed reaction [40]: (i) by changing the structure or dynamics of the enzyme’s binding site; (ii) by competitive binding of the organic solvent molecules; (iii) by removal of protein-bound water; and (iv) by improving the solubility of hydrophobic substrates. The solubilities of hydrophobic substrates are hard to calculate reliably in the MAOS system. Thus, they were not considered in this study. Combined with the points above, the distinction between lipases in MAOS systems and non-aqueous organic solvents/aqueous systems might be caused by structural changes and the positions of the protein-bound organic solvent molecules and water molecules.

### 3.2. The Property of the Lipase Surface

The property of the lipase surface is a crucial factor determining the position of organic solvent molecules and protein-bound waters. The solvent-accessible hydrophilic (SA_philic_) and hydrophobic (SA_phobic_) surface areas were calculated during the lipase simulations, including SA_philic_ and SA_phobic_ of CALB in various systems (Figure S1), CRL in various systems (Figure S2), and RCL in various systems (Figure S3). For CALB systems, hydrophilic surfaces show higher coverage than hydrophobic surfaces in the presence of n-heptane solvents during the simulations, whereas the opposite occurs in the MAOS and the aqueous systems. Notably, the total solvent accessible surface area (SASA) observed in the aqueous system was higher than that in the non-aqueous systems. The total SASA increased over simulation time in the MAOS system. Similarly, the CRL systems showed a total SASA in aqueous systems higher than those in the non-aqueous systems. In the CRL systems, the hydrophilic surfaces showed coverages higher than those of hydrophobic surfaces in all five systems (Figure S2). The RCL systems also exhibited trends similar to those of CRL (Figure S3).

The results of SASA represent tuning of the lipase systems to the properties of the solvent environment. In the crystal structure of CALB, the ratio (R_CALB_) of SA_philic_ and SA_phobic_ is about 1.20 (The SA_philic_ of the crystal structure of CALB is 65.9 nm^2^ and the SA_phobic_ is 54.7 nm^2^). At the initial stage of SA_philic_ and SA_phobic_ of CALB, they were close to each other; thus, after regulated in different systems, as expected in water and MAOS systems, the hydrophilic surfaces of CALB showed coverages higher than those of the hydrophobic surfaces, while in the non-aqueous organic solvent system the hydrophobic surfaces of CALB showed coverages higher than those of the hydrophilic surfaces. For both CRL and RCL crystal structures, the surface area of hydrophilic residues is significantly larger than the surface area of hydrophobic residues (R_CRL_ = 2.00, R_RCL_ = 2.24). Although the surface residue properties of both lipases were also changed under different solvent environments. For CRL (Figure S2), the surface area of hydrophilic residues is ~100 nm^2^ in organic solvents, ~110 nm^2^ in MAOS system, ~120 nm^2^ in water system, respectively. For RCL (Figure S3), the surface area of hydrophilic residues is ~70 nm^2^ in organic solvents, ~85 nm^2^ in MAOS system, ~95 nm^2^ in water system, respectively. The difference in the initial structures of both lipases resulted in a larger coverage area of hydrophilic residues than hydrophobic residues in organic solvents. For both CRL and RCL the change trend of SA_philic_ and SA_phobic_ from water to the MAOS systems or the non-aqueous organic solvent system is similar to CALB. The surfaces of these three lipases were smoother (or had a smaller protein radii) in the non-aqueous systems than in the other systems. A notable difference in SASA values was observed between non-aqueous systems and aqueous systems. Although the MAOS systems contained a small amount of water (3.5%, *v*/*v*), the lipases were clearly affected by the presence of the water. More details on the lipase-surface change were provided in Figures S4–S6 and the panoramic presentation of the hydrophilic and hydrophobic surfaces of lipase at 200 ns in various systems were provided in video 1, video 2 and video 3. In this process, a water shell formed on the lipase surface in the MAOS systems. We were curious about why such a small amount of water was so important to the structural changes of lipases, particularly in molecular dynamics simulation systems.

### 3.3. The Dynamic Behavior of Water in MAOS Systems

**Figure 3:**
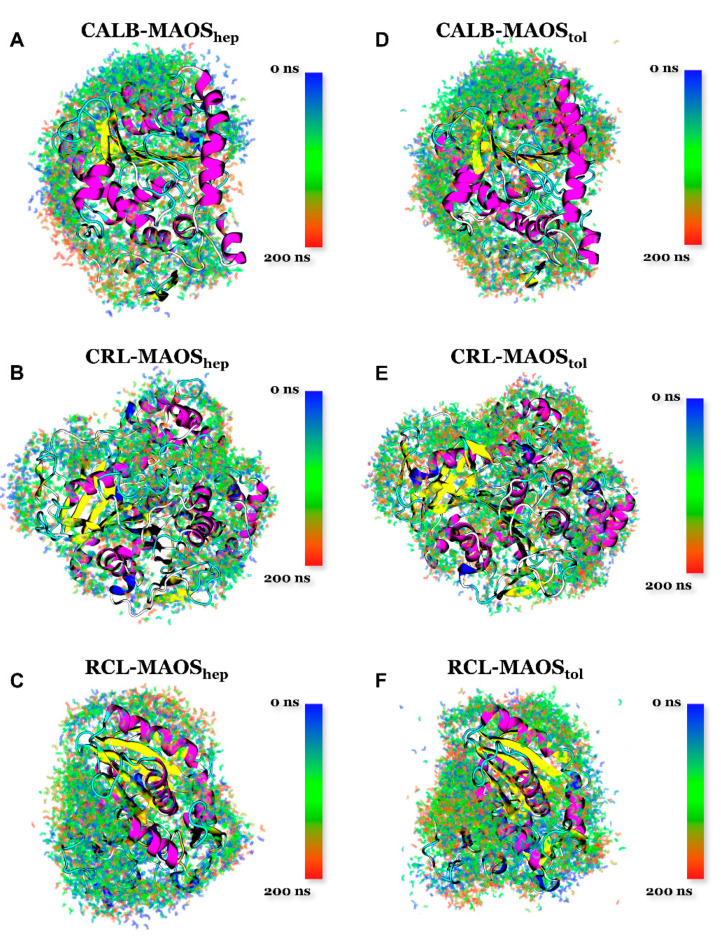
Snapshots of water at different instants in CALB-MAOS_hep_ systems (**A**), CRL-MAOS_hep_ systems (**B**), RCL-MAOS_hep_ systems (**C**), CALB-MAOS_tol_ (**D**), CRL-MAOS_tol_ (**E**), RCL-MAOS_tol_ (**F**). The time scale ranges from 0 ns to 200 ns and this corresponds to blue, green and red colors. Cartoon models of lipase with different colors represent different secondary structures; purple, blue, red, yellow, cyan and white parts represent alpha-helix, 3/10 helix, pi_helix, beta-sheet, turn and coil, respectively. More details of the lipase surface and snapshots of the waters in the MAOS systems are provided in the supplemental video.

We next investigated the water-binding position changes as a function of simulation time. Snapshots of the waters around the three lipases in the MAOS_hep_ and MAOS_tol_ systems at different instants were mapped in Figure 3. Hydration water formed water networks around the lipase surface to keep lipase in a “water-like” solution. Most of the water molecules accumulated in the hydrophilic surfaces, while a portion of the protein surface had no distribution of water molecules. The water molecules changed position over the simulation time (from 0 ns to 200 ns, snapshots of water from blue to red). In the snapshots in Figure 3, only a portion of the lipase surface and snapshots of the waters can be observed. Hence, more details of the lipase surface and snapshots of the waters in the MAOS systems were provided in supplemental video 4. The water-protein radial distribution functions (RDF) in the MAOS systems were calculated from the lipase simulations (Figure S7). The calculated water-protein RDF showed two maxima, representing two water shells around lipases at radial distances of 3.75 Å and 8.5 Å. The first shell consisted of water molecules with hydrogen bonds to the polar residues (or charged residues) of the lipases and the second shell might consist of water molecules with van der Waals interactions with the n-heptane molecules bound to the lipase surface. The first shell was much denser than the second shell because polar and charged residues predominated on the solvent accessible surface of proteins. On the other hand, the water shell on the lipase surface was more easily maintained in hydrophobic solvents (for example, n-heptane and toluene) than in hydrophilic organic solvents. This might explain why lipases were biologically inactivated in some hydrophilic organic solvents. In addition, the lipase in the MAOS systems increased the surface area of hydrophilic residues by ~10 nm^2^ compared to that in organic solvents (Figures S1–S3). Compared to the lipase in pure water, the surface hydrophilic residues area of lipase in the MAOS systems decreases by ~10 nm^2^. The behavior of lipase surface residues (including lid region, propeptide region and active pocket) is important for the function of lipase. According to previous study [41], dynamic flipping of hydrophobic phenylalanine located on the RCL surface affects the interfacial activation of lipase. In the primary analysis, the distribution and dynamic behavior of water molecules in the MAOS systems might selectively change the structures of lipases.

**Figure 4:**
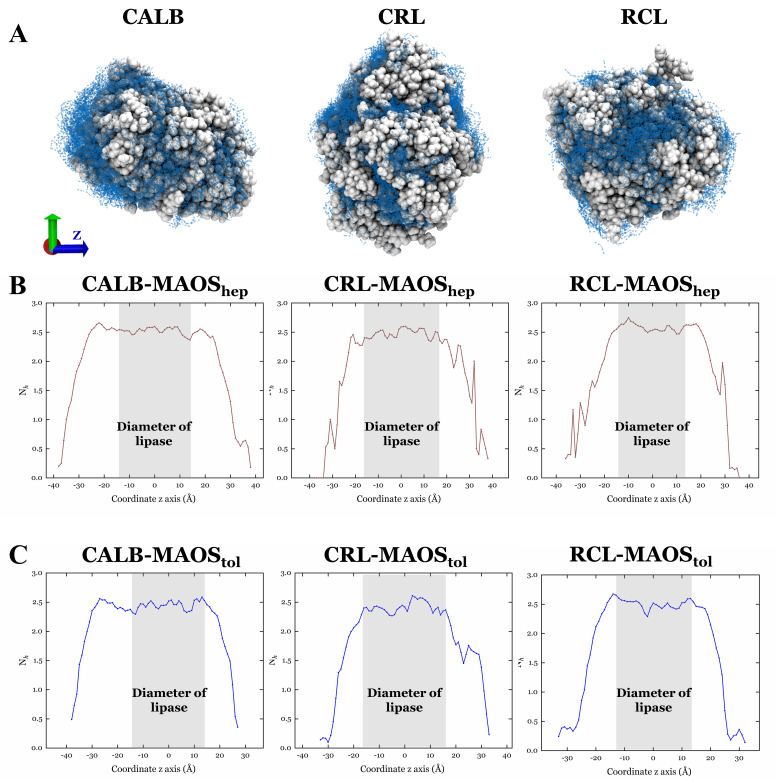
Hydrogen bonds formed by water molecules and the lipases in the MAOS systems (**A**). Distribution of hydrogen bonds formed by water molecules along the *z*-axis in the MAOS_hep_ (**B**) and MAOS_tol_ (**C**).

Hydrogen bonds (HBs) are major interactions between lipase and water. Likewise, the main reason for the unique properties of water as a solvent is its ability to form a spatially dynamic network of HBs. Computer simulations are widely used to investigate the HBs of water under different conditions. In the lipase-MAOS systems, the HBs affect the dynamic behavior of water molecules along with the surface properties of the protein. The in-depth information on the dynamics of HBs and their lifetimes can be used to provide insights into the dynamics of the water molecules in the MAOS systems. The lifetimes of the HBs was shown in Table S2. It showed picosecond-scale lifetimes of HBs in a variety of systems. The value is reasonable compared to the experimental result on relaxation times for different physical properties of water, for example, its vibrational relaxation times (0.74 ps [42]) and the rotational relaxation times (0.6 ps [43]). The results of HBs lifetimes indicated that the HBs between water molecules in the MAOS media were more stable than those between water molecules in lipase-water systems. Most of the substrates for the ester synthesis reaction of lipases, such as ethanol and octanoic acid, are water-soluble. The products of ester synthesis reactions of lipases are often insoluble in water, such as ethyl octanoate. The water shell around the lipase in the MAOS systems might help stabilize the substrate around the lipases which appears to facilitate the ester synthesis reaction much more readily than those in pure water. The HBs lifetimes in the MAOS systems with n-heptane was also different from that with toluene. It was hypothesized that the different polarity indexes of organic solvents in the MAOS media and the number of water molecules (600 in MAOS_hep_ and 414 in MAOS_tol_) are responsible for this difference in the MAOS systems. Meanwhile, the HBs number of lipase-MAOS systems was counted as a function of the z-axis sections displayed in Figure 4. The average number of hydrogen bonds in various systems per water molecule was shown in Table S3. The regions where the hydrogen bonds were mainly distributed in the lipase-MAOS systems stabilized surround the protein according to the size of various lipases.

The mean-square displacement (MSD) function is a powerful tool used in tracking atomic motion in large complicated systems. The MSD curves for water molecules in the MAOS systems were reported in Figure S8. From the MSD curves, average diffusion coefficients were calculated according to the Einstein relation [44]. The diffusion constants of water molecules in MAOS_hep_ systems were distinct from those in MAOS_tol_. The diffusion constants of water molecules in MAOS media ranged from 0.13 × 10^−5^ cm^2^/s to 0.72 × 10^−5^ cm^2^/s, which were significantly smaller than the experimental value of the diffusion constant of pure water (2.29 × 10^−5^ cm^2^/s). The rotation correlation function (RCF) of solvation shell waters in different systems was shown in Figure S9. The RCF decayed mainly through the overall water molecular rotation during the simulation. It was observed a lower rotation rate of water in the MAOS systems than that of water in pure water systems. Taken with the association findings discussed in the preceding paragraph, this also illustrated the presence of a stable water shell around the lipase in the MAOS media.

Generally, hydrophobic solvents have more difficulty stripping essential water from enzyme molecules than hydrophilic solvents. As in a previous study [45], two major water shells were observed around the lipases in the MAOS systems. Although the water shell in MAOS is discontiguous and does not cover the entire area of the protein surface, it still has properties similar to those of aqueous solutions. The water shell bound to the protein is important for protein function and retaining the 3D structure and activity, as it represents an integral part of the protein structure [46]. However, in the presence of organic solvents alone, the position of the hydration shell or layer of water is displaced by organic solvent, which results in a dramatic change in protein structure, deformation of the active site geometry and a resulting change in activity. This is perhaps the reason for the difference between MAOS systems and organic solvent systems.

### 3.4. The Energy Landscape and the Minimum Energy Path in the Presence of Different Solvents

**Figure 5:**
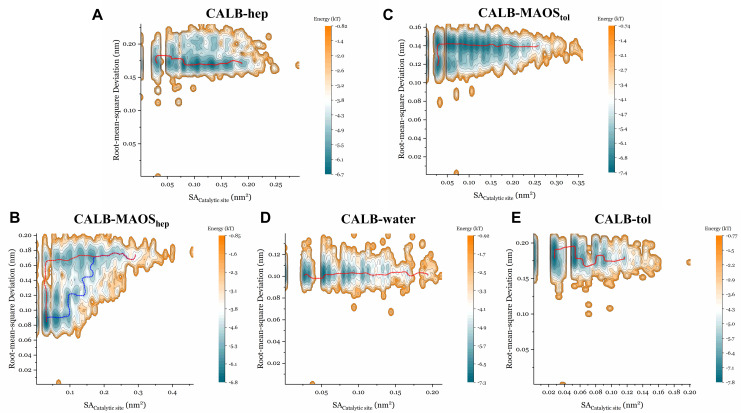
Energy landscape analysis of CALB with correlation to RMSD and the solvent accessible area of catalytic residues in the n-heptane systems (**A**), in the MAOS_hep_ system (**B**), in the MAOS_tol_ system (**C**), in water (**D**) and in the toluene system (**E**). Energy E(x) was calculated through the Boltzmann distribution. Energy landscape analysis was conducted according to the probability density P(x). Each trajectory was sampled every 100 ps in the EL analysis. The red and blue lines represent the minimum energy path.

**Figure 6:**
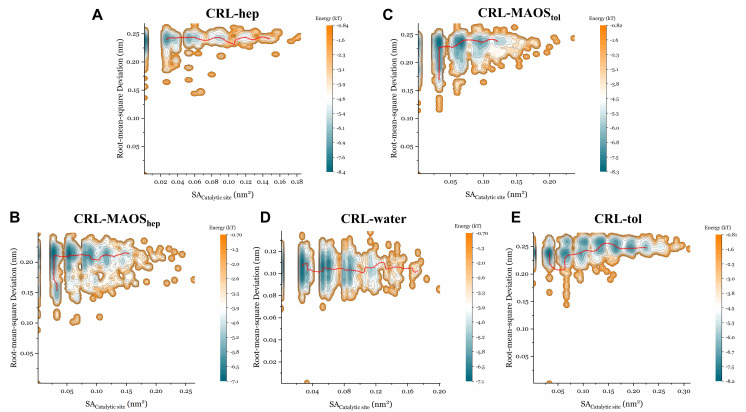
Energy landscape analysis of CRL with correlation to RMSD and the solvent accessible area of catalytic residues in the n-heptane system (**A**), in the MAOS_hep_ system (**B**), in the MAOS_tol_ system (**C**), in water (**D**) and in the toluene system (**E**). Energy E(x) was calculated through the Boltzmann distribution. Energy landscape analysis was conducted according to the probability density P(x). Each trajectory was sampled every 100 ps in the EL analysis. The red line represents the minimum energy path.

**Figure 7:**
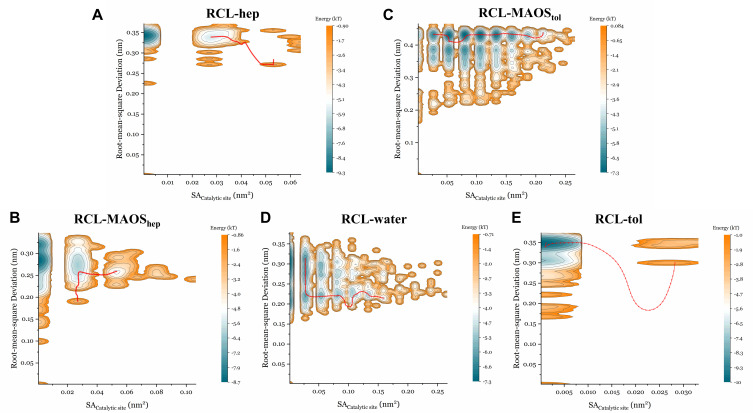
Energy landscape analysis of RCL with correlation to RMSD and the solvent accessible area of catalytic residues in the n-heptane system (**A**), in the MAOS_hep_ system (**B**), in the MAOS_tol_ system (**C**), in water (**D**) and in the toluene system (**E**). Energy E(x) was calculated through the Boltzmann distribution. Energy landscape analysis was conducted according to the probability density P(x). Each trajectory was sampled every 100 ps in the EL analysis. The red line represents the minimum energy path.

We next plotted the energy landscapes (EL) along RMSD and solvent accessible areas of catalytic residues (SA_Catalytic__site) sampled by MD simulations and identified the minimum energy paths (MEP) shown in Figure 5 (CALB), Figure 6 (CRL) and Figure 7 (RCL). Researchers have explored a wide variety of models based on energy landscape theory to investigate various biomolecular dynamics such as protein conformational changes [47,48]. For enzymes, the energy landscape is very complex and it is difficult to precisely describe the overall motion of the protein. It is well known that the catalytic site of lipases is characterized by the so-called catalytic triad composed of Ser, His and Asp. Ser is involved in covalent catalysis and His activates Ser to undergo nucleophilic attack [49]. Exposure of Ser is a prerequisite for the lipase catalytic reaction and this is the main reason for plotting the energy landscapes along SA_Catalytic__site. Rigorous calculation of a 2D free energy surface for this system is time-consuming and difficult. In the present study, only relative free energies were considered and the global minimum was used as the reference to compute the relative free energies. The range of relative free energy calculation in this study (∼5 kcal/mol) is reasonable compared to the range of relative free energy calculation in other publications [50].

For CALB systems, RMSD values in n-heptane systems (Figure 5A) were clustered at approximately 0.18 Å and the SA_Catalytic_site_ values were mainly distributed in the range 0.02 nm^2^ to 0.2 nm^2^. The detailed energy along the MEP was provided in Figure S7. The point with the highest energy along the MEP was the transition state (TS). The values of SA_Catalytic_site_ in the n-heptane (Figure 5A) systems were clustered at 0.08 nm^2^, 0.105 nm^2^ and 0.135 nm^2^. The RMSD values in the MAOS systems (Figure 5B) were distributed in the range 0.08 Å to 0.17 Å and the SA_Catalytic_site_ values were mainly distributed in the range of 0.03 nm^2^ to 0.3 nm^2^. Notably, two major MEPs were observed in the MAOS systems. The energy along the MEP in the MAOS systems (Figure S10B) indicated that MEP2 (the blue path in Figure 5B) has significantly lower barriers than MEP1 (the red path in Figure 5B). The RMSD analysis (Figure S11A) demonstrated that the RMSD values increased over the simulation time in the MAOS systems. Together, the values of SA_Catalytic_site_ and MEP2 in the MAOS systems showed greater exposure of catalytic Ser during the later stages of the simulations. The RMSD values in the presence of water (Figure 5D) were clustered at approximately 0.10 Å and the SA_Catalytic_site_ values were mainly distributed in the range 0.03 nm^2^ to 0.15 nm^2^. The energy along the MEP in the aqueous systems showed a higher energy barrier than those of other systems. This might imply that the CALB had more difficulty switching between open and closed conformations in aqueous systems.

For the CRL system, RMSD values in the n-heptane systems (Figure 6A) were clustered at approximately 0.24 Å and the SA_Catalytic_site_ values were mainly distributed in the range 0.03 nm^2^ to 0.12 nm^2^. The energy along the MEP (Figure S7D) in the n-heptane system indicated that the values of SA_Catalytic_site_ were clustered at 0.05 nm^2^, 0.065 nm^2^ and 0.085 nm^2^. The RMSD values in the MAOS_hep_ systems (Figure 6B) were distributed in the range 0.15 Å to 0.2 Å and the SA_Catalytic_site_ values were mainly distributed in the range 0.025 nm^2^ to 0.17 nm^2^. The energy along the MEP (Figure S10E) in the MAOS systems indicated that the values of SA_Catalytic_site_ were clustered at 0.028 nm^2^, 0.055 nm^2^ and 0.090 nm^2^. The proportion of open conformations for CRL in the MAOS was greater than that in the n-heptane systems. The RMSD values in the aqueous systems (Figure 6D) were clustered at approximately 0.1 Å and the SA_Catalytic_site_ values were mainly distributed in the range 0.03 nm^2^ to 0.12 nm^2^. As with the CALB systems, the CRL also showed a difficult switch between open and closed conformations in the aqueous systems, according to the energies calculated along the MEP (Figure S10F).

For RCL systems, RMSD values in the n-heptane systems (Figure 7A) were clustered at approximately 0.35 Å and the SA_Catalytic_site_ values were mainly distributed in the range 0 nm^2^ to 0.03 nm^2^. The RMSD values in the MAOS_hep_ systems (Figure 7B) were clustered in the range 0.2 Å to 0.32 Å and the SA_Catalytic_site_ values were mainly distributed in the range 0 nm^2^ to 0.05 nm^2^. The energies along the MEPs in the n-heptane and MAOS systems both revealed very high energy barriers and it was difficult for the RCL to switch between open and closed conformations in the n-heptane and MAOS systems. The catalytic Ser in most conformations in the n-heptane and MAOS systems was insufficiently exposed. The values of the RMSD in the aqueous systems (Figure 7D) were clustered in the range 0.2 Å to 0.3 Å and the values of SA_Catalytic_site_ were mainly distributed in the range 0.03 nm^2^ to 0.16 nm^2^. The conformations of the TS in aqueous systems indicated a more exposed catalytic Ser conformation during the simulation.

In the previous study, we demonstrated with simulations and experimental results that the fast flipping rate from the closed state to the open state is the main reason for lipase activation [41]. Exposure of Ser is a prerequisite for hydrolysis and esterification reactions. Among the five lipase systems, the lipases in the MAOS systems exhibited distinct differences from the others; these differences included the exposures of catalytic residues and the energies along the MEP. The results of the previous section suggest that the discontiguous water shells in the MAOS systems might be one of the major causes accounting for these discrepancies.

### 3.5. The Effect of Water Content on Lipase Synthetic Activities

To evaluate the effect of water content on the synthetic activity of the lipase, the experiments of RCL synthetic reaction with a gradient of water content were performed (Figure S12). It indicated that the synthetic activity of RCL in non-aqueous organic solvent media was significantly lower than that in MAOS media (water content 3.5%, *v*/*v*). Similar to the previous study, *Pseudomonas cepacia* lipase in non-aqueous media showed a lower synthetic activity than that in MAOS media [51]. The presence of small amounts of water molecules is important for the synthesis reaction of lipase. Some investigators believe that there is a minimum and optimum water level required for providing enough conformational flexibility for lipase in non-water systems [51]. However, as water content increases in Figure S12, the hydrolysis started competing with the transesterification as the equilibrium shifted towards the hydrolysis. The synthetic activity of RCL in system with 20% water content was also significantly lower than that in MAOS media. As discussed in previous sections, with the increase of water content in the system, more water molecules may be aggregated in the hydrophobic surface of lipase, which makes the conformation of lipase in this system different from that in MAOS systems. The conformation of the lipase in the system with 20% or higher water content might be more suitable for hydrolysis.

Overall, the lipases in the MAOS systems showed unique conformations different from those of the non-aqueous organic solvent systems and aqueous systems. The position of water molecules aggregated on the lipase surface in MAOS media might the major determinant of the unique conformations of lipases and it particularly impacted the distribution of hydrophilic and hydrophobic amino acids on the lipase surface. The water molecules in the MAOS systems form two water shells around the lipase and these exhibit dynamic behavior similar to those operating in water systems. The energy landscapes of the lipases along the RMSD/SA_Catalytic_site_ and the MEP indicated that the dynamic open states of the lipases in the MAOS systems differed from those in other solvent environments. To more accurately simulate lipase in organic media, the microenvironment (for example, a small amount of water in organic media) of a real organic solvent system should be seriously considered. This study confirms the necessity of paying attention to the microenvironment in MD simulations of lipase-catalyzed reactions in organic media.

## Supplementary Materials

The following are available online at https://www.mdpi.com/article/10.3390/biom11060848/s1, Figure S1–S3: The SASA of lipases in various systems, Figure S4–S6: The hydrophilic and hydrophobic surfaces of lipases at different instants in various systems, Figure S7: Water-lipase RDF in various systems, Figure S8: The MSD function of water molecules in various systems, Figure S9: The RCF of water molecules in various systems, Figure S10: The energy of lipases along the MEP in various systems, Figure S11: The RMSD of lipase in various systems, Figure S12: Relative activity of lipase synthesis in systems with different water contents, Table S1: Comparison of the experimental density with the simulated density of organic solvents used in simulation, Table S2: Hydrogen bonding lifetimes of H_2_O-H_2_O and Protein-H_2_O, Table S3: The average number of hydrogen bonds of H_2_O-H_2_O and Protein-H_2_O per water molecule, Video S1–S3: The panoramic presentation of the hydrophilic and hydrophobic surfaces of lipases in various systems, Video S4: The panoramic presentation of the snapshots of water at different instants in various systems.

## Abbreviations

MD	Molecular dynamic
CALB	*Candida antarctica* lipase B
CRL	*Candida rugosa* lipase
RCL	*Rhizopus chinensis* lipase
MAOS	Micro-aqueous organic solvents
RDF	Radial distribution function
RMSF	Root-mean-square fluctuation
RMSD	Root-mean-square deviation
SASA	Solvent accessible surface area
RCF	Rotation correlation function
MSD	Mean-square displacement
HBs	Hydrogen bonds
EL	Energy landscapes
FID	Flame ionization detector
MEP	Minimum energy path
PCM	Polarizable continuum model
SMD	Solvation Model Based on Density
GAFF	General Amber force field
