# Supplementary material for: Micro-Aqueous Organic System: A Neglected Model in Computational Lipase Design?
Source: Biomolecules. 2021 Jun 7;11(6):848. doi: 10.3390/biom11060848 (PMC8226779; doi:10.3390/biom11060848)
Supplement: Supplementary file 1 [file biomolecules-11-00848-s001.zip › Supplementary Materials.pdf]

Supplementary Materials

Micro-aqueous organic system: A neglected model in computational lipase design?

Shang Wang[1], Yan Xu[1], and Xiao-Wei Yu[1,*]

[1] Key Laboratory of Industrial Biotechnology, Ministry of Education, School of Biotechnology, Jiangnan University, Wuxi 214122, PRC

[*] Corresponding author

e-mail: yuxw@jiangnan.edu.cn

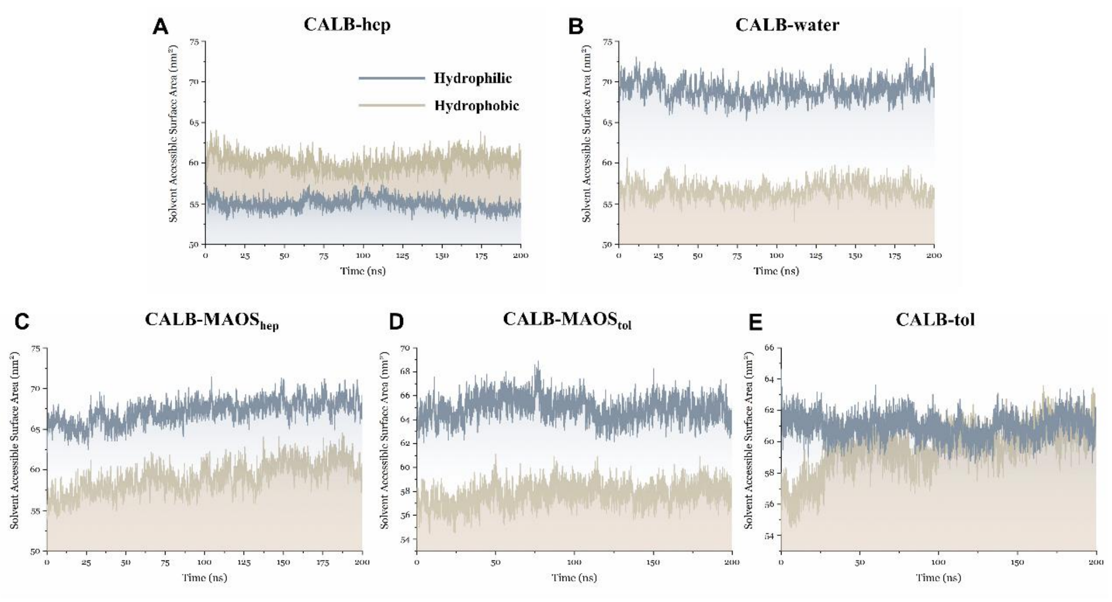

Figure S1. The solvent accessible surface area (SASA) of CALB in n-heptane system (A), water system (B), MAOS$_{hep}$ system (C), MAOS$_{tol}$ system (D), and toluene system (E).

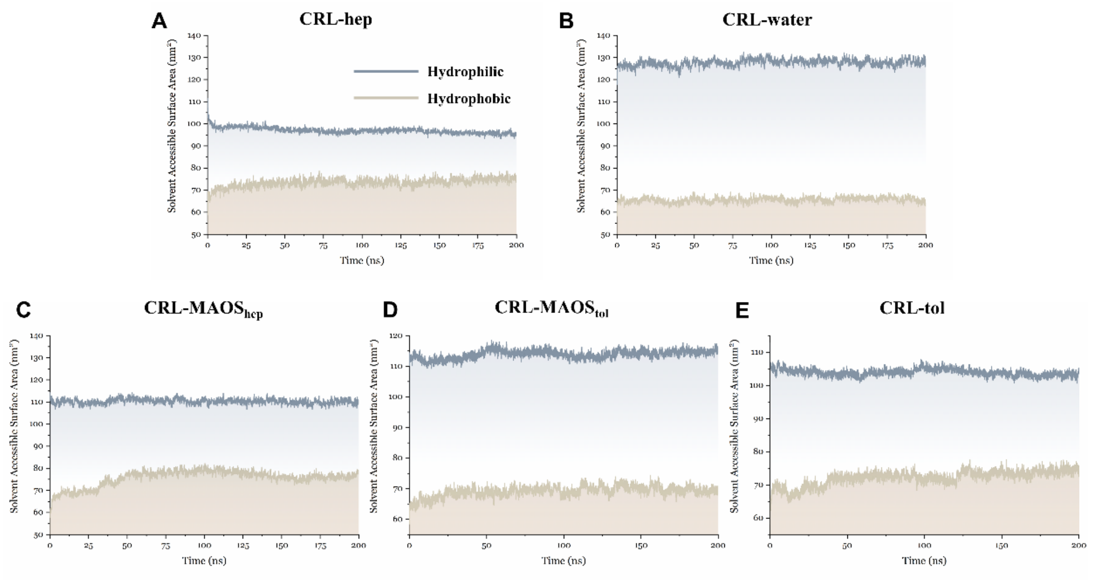

Figure S2. The solvent accessible surface area (SASA) of CRL in n-heptane system (A), water system (B), MAOS$_{hep}$ system (C), MAOS$_{tol}$ system (D), and toluene system (E).

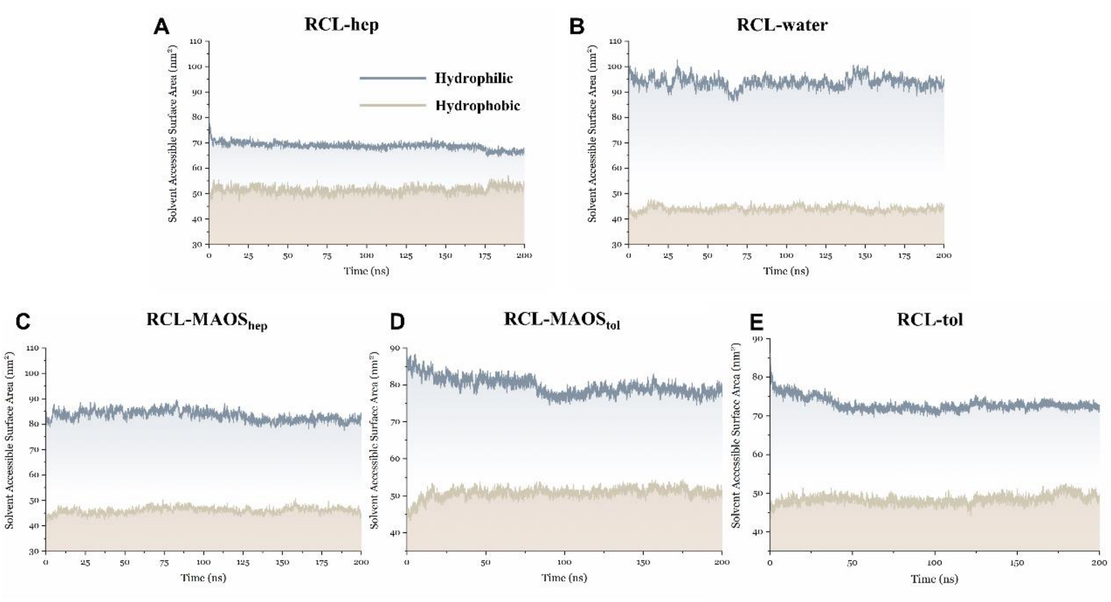

Figure S3. The solvent accessible surface area (SASA) of RCL in n-heptane system (A), water system (B), $MAOS_{hep}$ system (C), $MAOS_{tol}$ system (D), and toluene system (E).

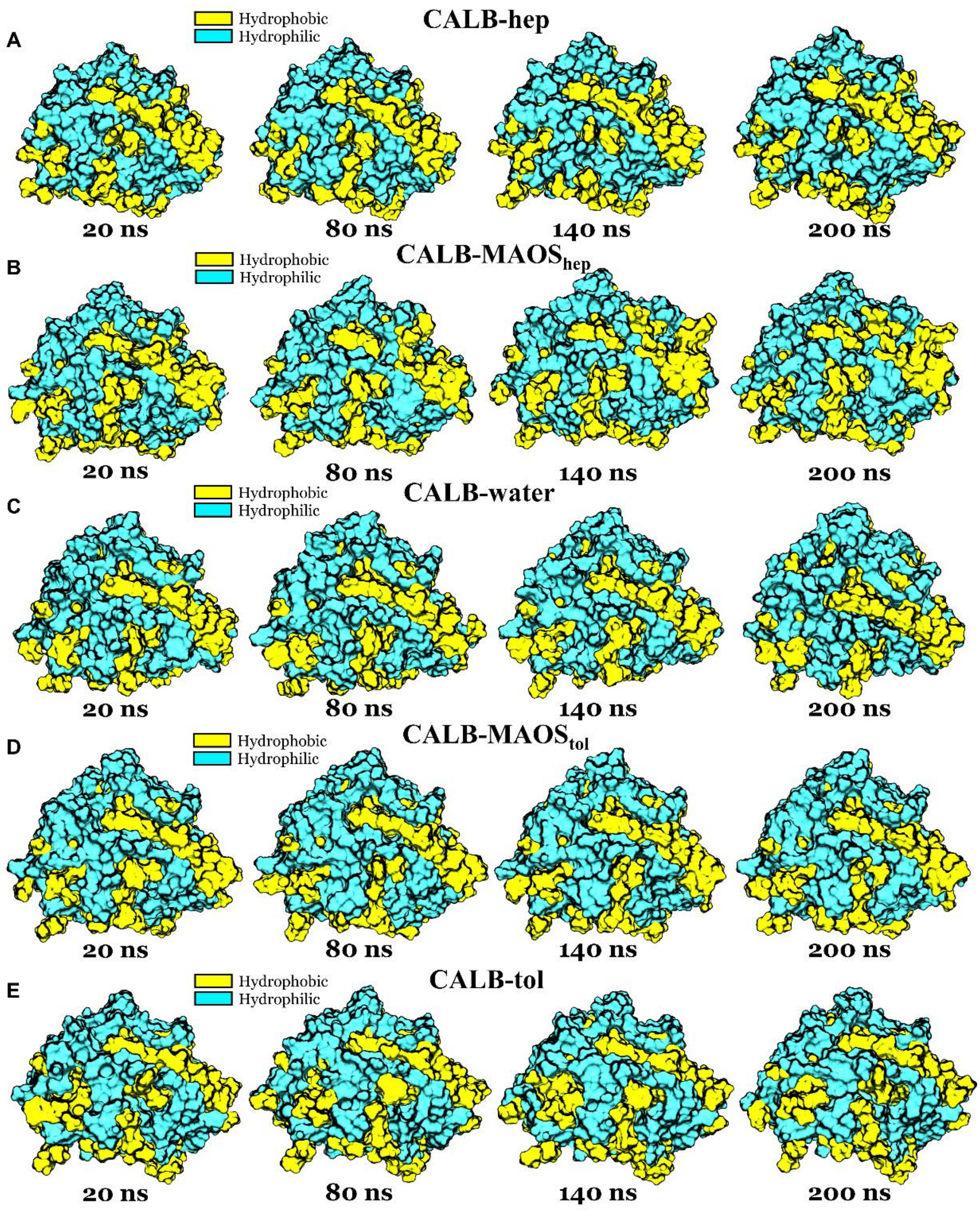

Figure S4. The hydrophilic (cyan) and hydrophobic (yellow) surfaces of CALB at different instants (20 ns, 80 ns, 140 ns, 200 ns) in n-heptane systems (A), MAOS$_{hep}$ systems (B), water systems(C), MAOS$_{tol}$ systems (D), and toluene systems (E). More details about hydrophilic and hydrophobic surface residues of CALB were provided in video 2.

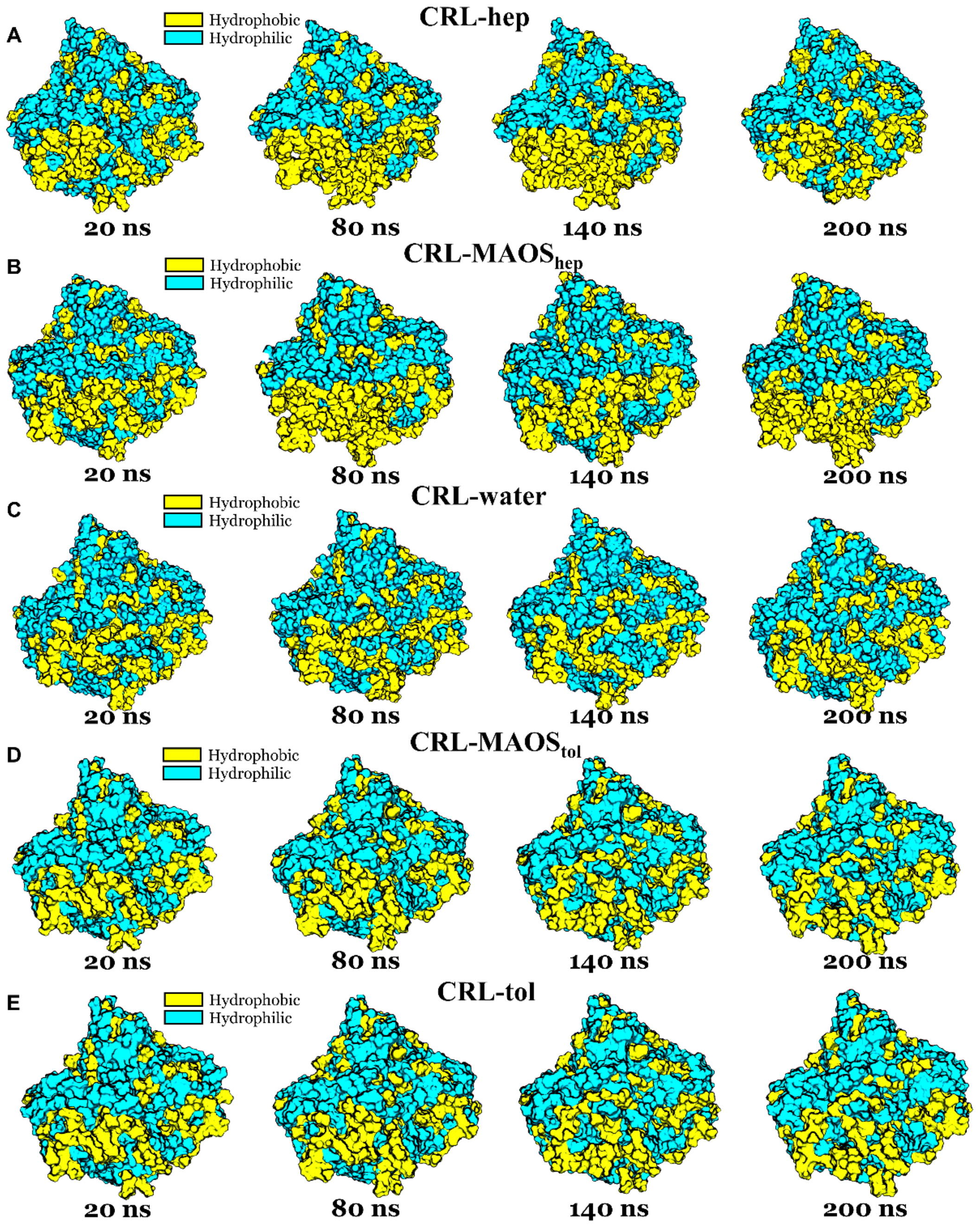

Figure S5. The hydrophilic (cyan) and hydrophobic (yellow) surfaces of CRL at different instants (20 ns, 80 ns, 140 ns, 200 ns) in n-heptane systems (A), MAOS$_{hep}$ systems (B), water systems(C), MAOS$_{tol}$ systems (D), and toluene systems (E). More details about hydrophilic and hydrophobic surface residues of CRL were provided in video 3.

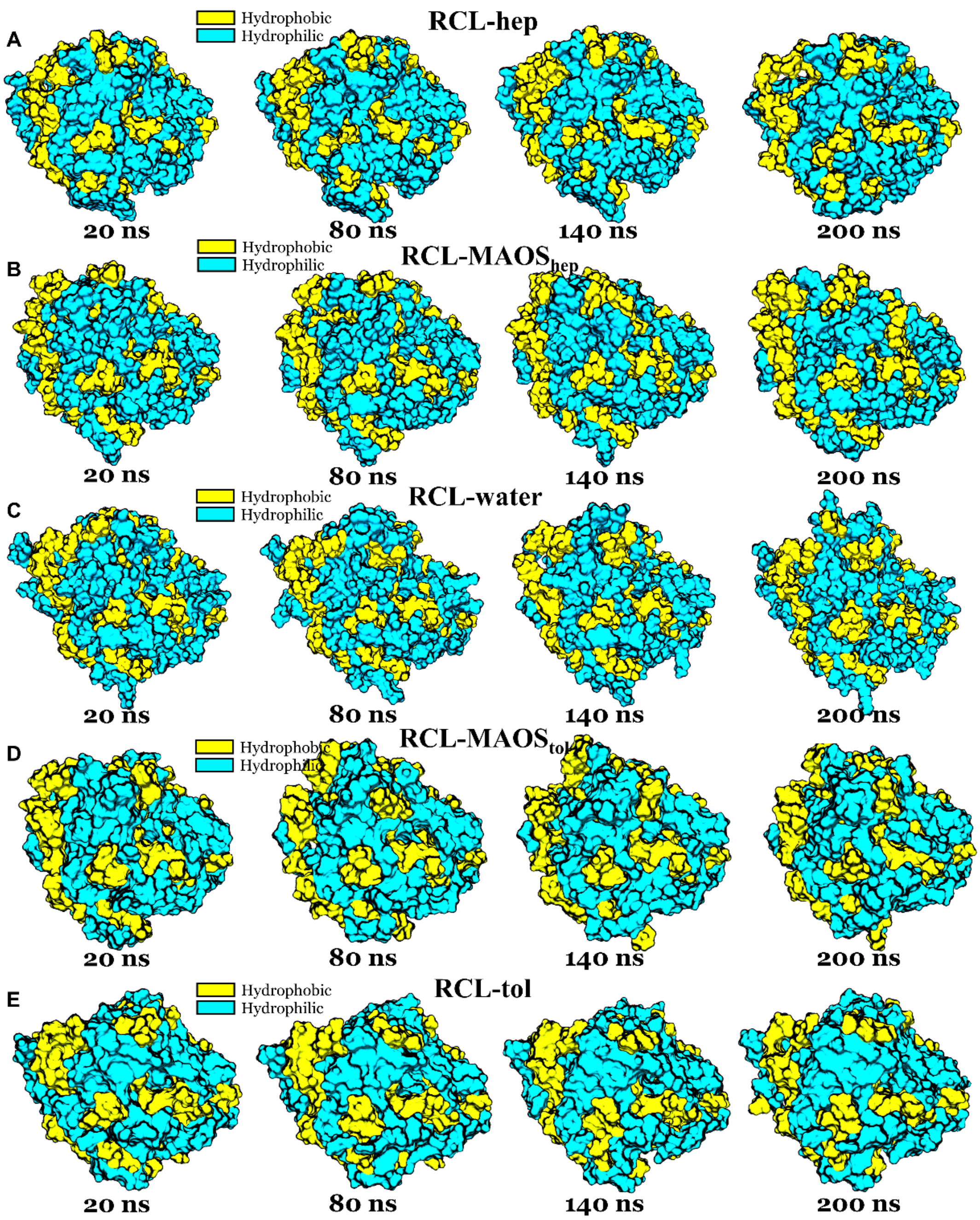

Figure S6. The hydrophilic (cyan) and hydrophobic (yellow) surfaces of RCL at different instants (20 ns, 80 ns, 140 ns, 200 ns) in n-heptane systems (A), $MAOS_{hep}$ systems (B), water systems(C), $MAOS_{tol}$ systems (D), and toluene systems (E). More details about hydrophilic and hydrophobic surface residues of RCL were provided in video 4.

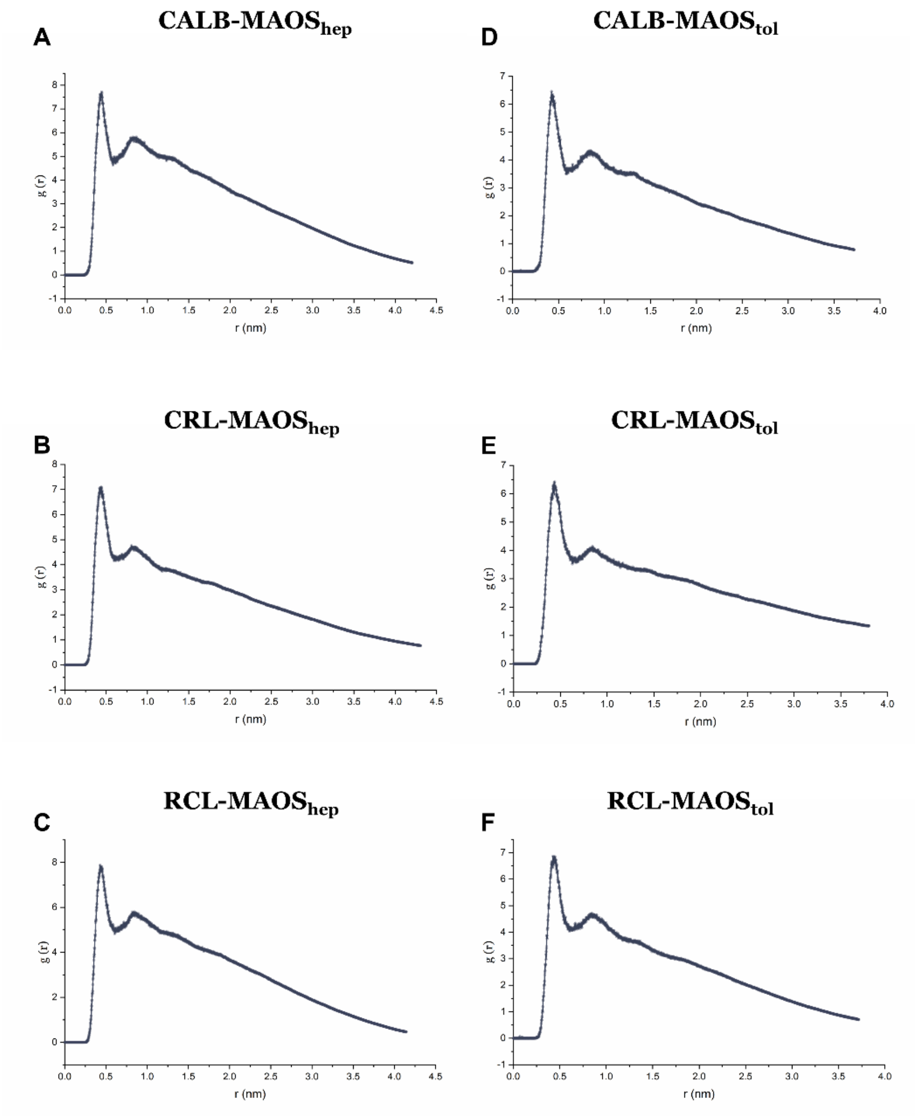

Figure S7. Water-lipase radial distribution functions are shown for CALB-MAOS$_{hep}$ systems (A), CRL-MAOS$_{hep}$ systems (B), RCL-MAOS$_{hep}$ systems (C), CALB-MAOS$_{tol}$ (D), CRL-MAOS$_{tol}$ (E), and RCL-MAOS$_{tol}$ (F).

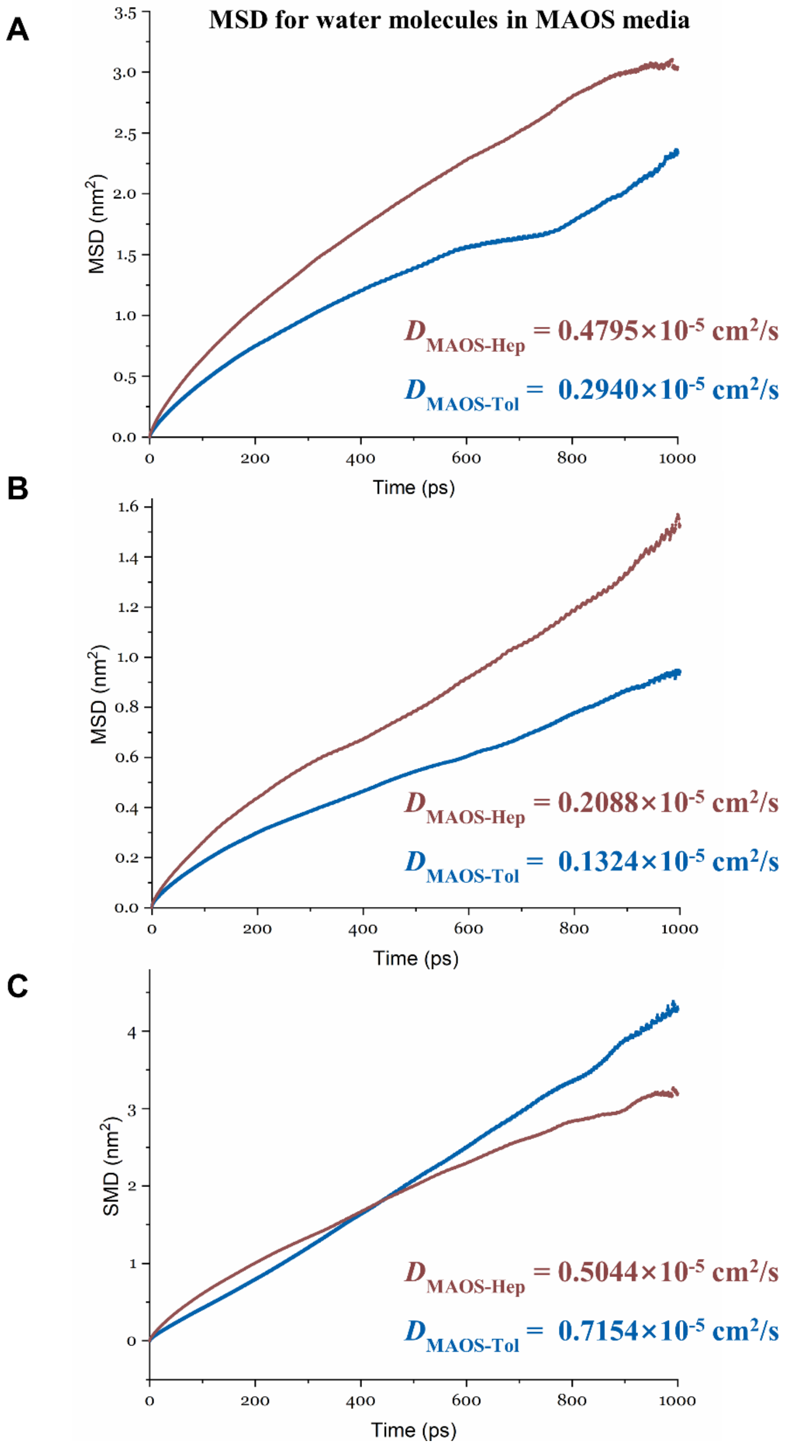

Figure S8. The mean-square displacement (MSD) function of water molecules in CALB-MAOS systems (A), CRL-MAOS systems (B), and RCL-MAOS systems (C). The diffusion constant of water in MAOShep (red) and MAOStol (blue).

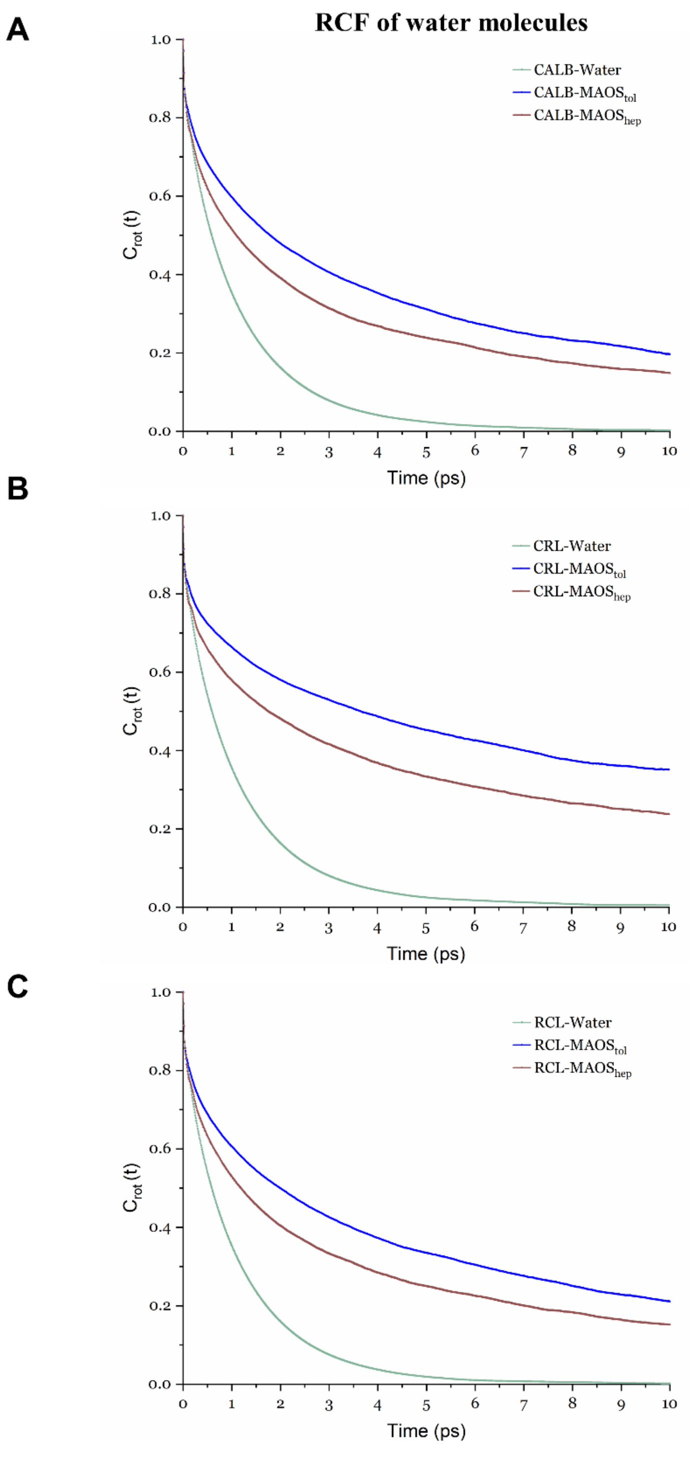

Figure S9. The rotation correlation function (RCF) of water molecules in CALB systems (A), CRL systems (B), and RCL systems (C).

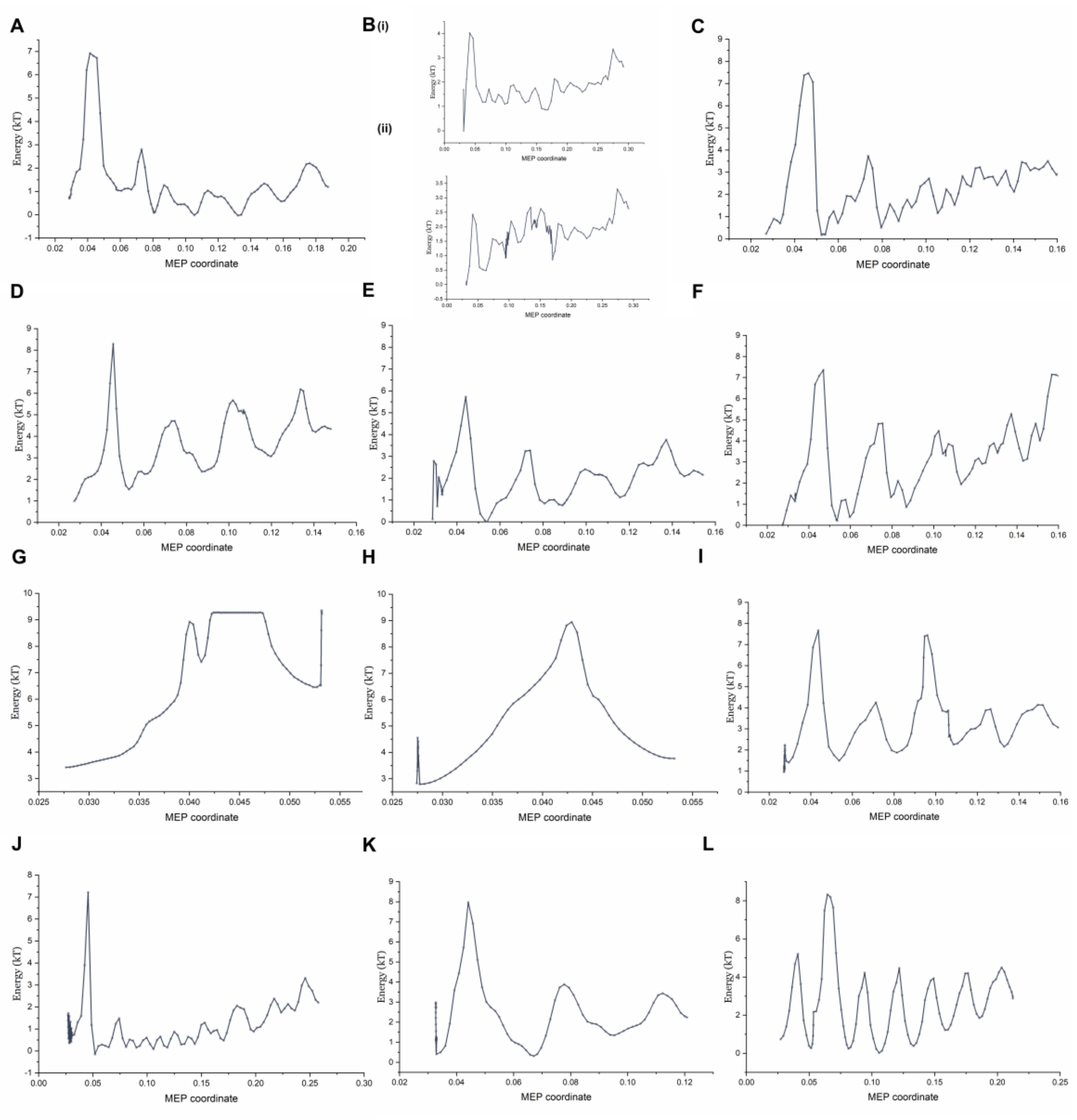

Figure S10. The energy of CALB along the minimum energy path (MEP) in the n-heptane systems (A), $MAOS_{hep}$ systems (B), water systems (C), and $MAOS_{tol}$ systems (J). The energy of CRL along the MEP in the n-heptane systems (D), $MAOS_{hep}$ systems (E), water systems (F), and $MAOS_{tol}$ systems (K). The energy of RCL along the MEP in the n-heptane systems (G), $MAOS_{hep}$ systems (H), water systems (I), and $MAOS_{tol}$ systems (L). The Figure S10B(i) represents the minimum energy path 1 and (ii) represents the minimum energy path 2. MEP coordinate represents the projection of MEP on the x-axis.

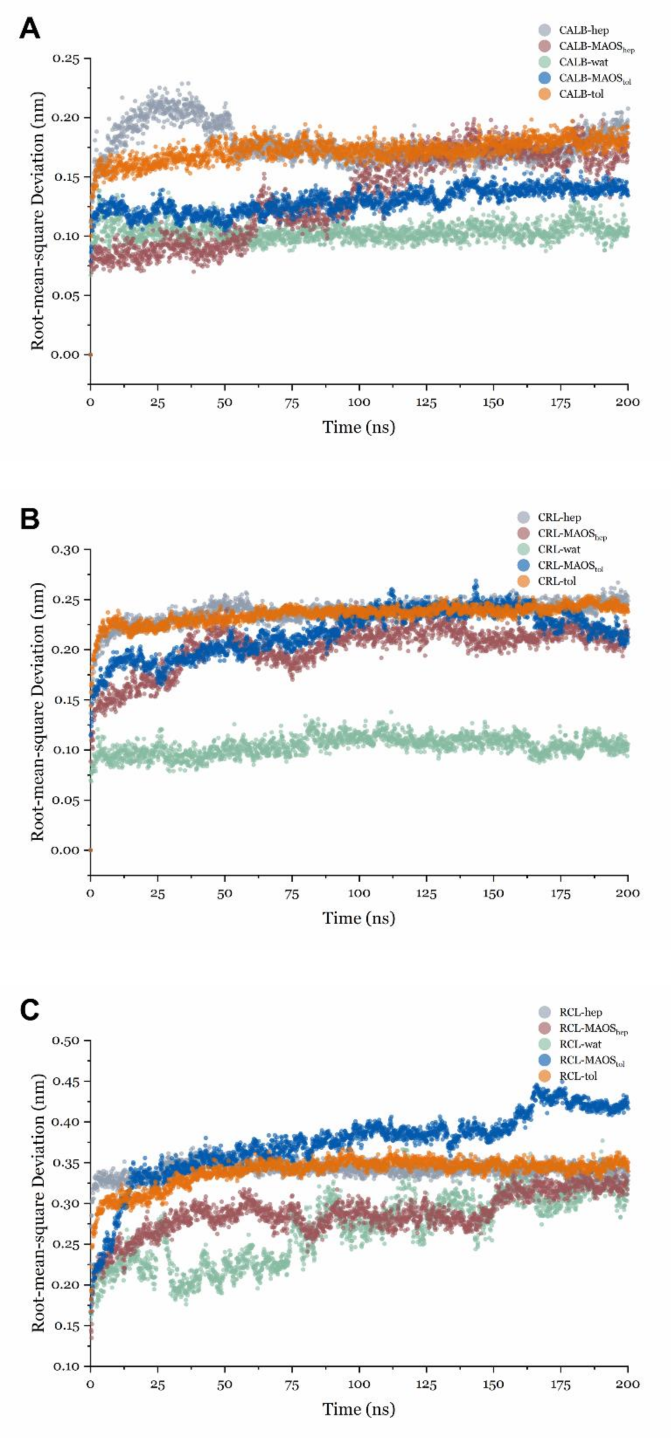

Figure S11. The RMSD of CALB (A), CRL (B), and RCL (C) in the in water (green), MAOShep (red), MAOStol (blue), n-heptane (ice-blue), toluene (orange).

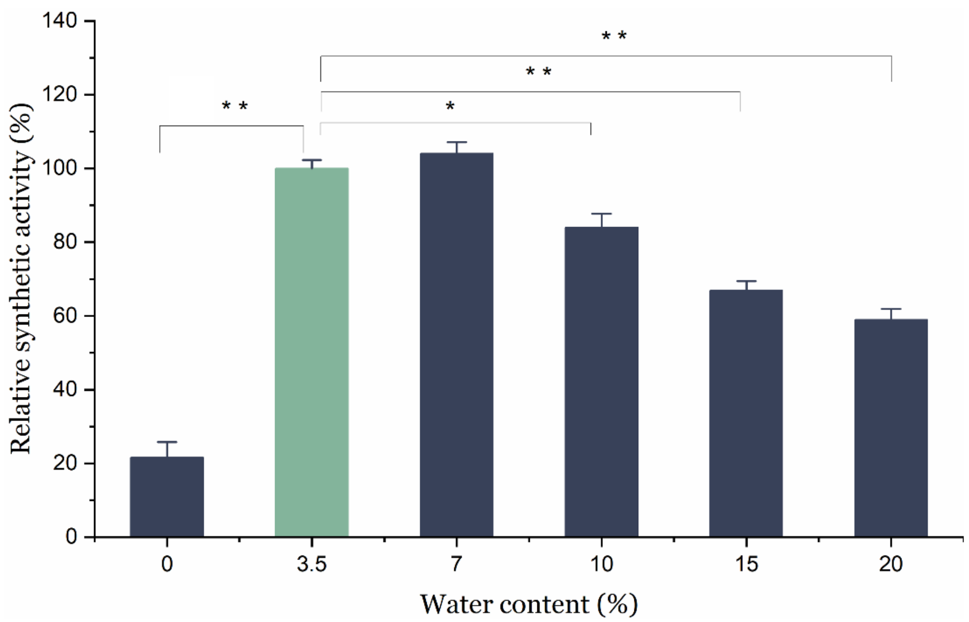

Figure S12. Relative activity of lipase synthesis in systems with different water contents (v/v). The synthetic activity of the control system (green, water content 3.5%, v/v) is set at 100%. The synthesis reaction substrate is octanoic acid and ethanol, the product is ethyl octanoate, and the solvent is n-heptane. * represents significant difference ($p < 0.05$), and ** represents significant difference ($p < 0.01$).

Table S1. Comparison of the experimental density with the simulated density of organic solvents used in simulation

	CALB-hep	CRL-hep	RCL-hep	CALB-tol	CRL-tol	RCL-tol
Experimental density[a] (g/cm^3)	0.68[a]	0.68[a]	0.68[a]	0.86[a]	0.86[a]	0.86[a]
Simulated density[b] (g/cm^3)	0.71[b]	0.73[b]	0.70[b]	0.88[b]	0.89[b]	0.88[b]

[a] Density of n-heptane solution at 25 °C

[b] Density of protein-heptane/toluene box at 40 °C

Table S2. Hydrogen bonding lifetimes of H_2O-H_2O and Protein-H_2O

System	Lipase	Life Time$_{H2O-H2O}$ (ps)	Life Time$_{H2O-Protein}$ (ps)
	CALB	0.54	0.71
**MAOS$_{hep}$**	CRL	0.58	0.72
	RCL	0.54	0.68
	CALB	0.63	0.77
**MAOS$_{tol}$**	CRL	0.69	0.77
	RCL	0.65	0.71
	CALB	0.45	0.67
**Water**	CRL	0.45	0.68
	RCL	0.45	0.64

Table S3. The average number of hydrogen bonds of H_2O-H_2O and Protein-H_2O per water molecule

System	Lipase	Number of HB$_{H2O-H2O}$	Number of HB$_{H2O-Protein}$
	CALB	1.12 ± 0.025	1.23 ± 0.071
**MAOS$_{hep}$**	CRL	0.76 ± 0.024	1.84 ± 0.087
	RCL	1.03 ± 0.023	1.45 ± 0.058
	CALB	0.91 ± 0.028	1.55 ± 0.075
**MAOS$_{tol}$**	CRL	0.62 ± 0.025	1.96 ± 0.098
	RCL	0.86 ± 0.027	`1.73 ± 0.072

Video Legends

Video 1. The panoramic presentation of the hydrophilic (cyan) and hydrophobic (yellow) surfaces of CALB at 200 ns in n-heptane systems, $MAOS_{hep}$ systems, water systems, $MAOS_{tol}$ systems, and toluene systems.

Video 2. The panoramic presentation of the hydrophilic (cyan) and hydrophobic (yellow) surfaces of CRL at 200 ns in n-heptane systems, $MAOS_{hep}$ systems, water systems, $MAOS_{tol}$ systems, and toluene systems.

Video 3. The panoramic presentation of the hydrophilic (cyan) and hydrophobic (yellow) surfaces of RCL at 200 ns in n-heptane systems, $MAOS_{hep}$ systems, water systems, $MAOS_{tol}$ systems, and toluene systems.

Video 4. The panoramic presentation of the snapshots of water at different instants in CALB-MAOS system, CRL-MAOS system, and RCL-MAOS system. The time scale ranges from 0 ns to 200 ns and this corresponds to blue, green, and red colors. Cartoon models of lipase with different colors represent different secondary structures; purple, blue, red, yellow, cyan, and white parts represent alpha-helix, 3/10 helix, pi_helix, beta-sheet, turn, and coil, respectively.
